# From In Vivo Predictive Dissolution to Virtual Bioequivalence: A GastroPlus^®^-Driven Framework for Generic Candesartan Cilexetil Tablets

**DOI:** 10.3390/ph18040562

**Published:** 2025-04-11

**Authors:** Hao Ruan, Xiaoting Geng, Zijing Situ, Qian Shen, Tianjian Ye, Xin Chen, Weike Su

**Affiliations:** 1 College of Pharmaceutical Sciences, Zhejiang University of Technology, Hangzhou 310014, China; clare_ruan@163.com; 2 Zhejiang Key Laboratory of Biopharmaceutical Contact Materials, NMPA Key Laboratory for Core Technology of Generic Drug Evaluation, Zhejiang Institute for Food and Drug Control, Hangzhou 310052, China; 3 School of Pharmacy, China Pharmaceutical University, Nanjing 210009, China; 4 Zhejiang Yongning Pharmaceutical Co., Ltd., Taizhou 318020, China

**Keywords:** candesartan cilexetil, GastroPlus^®^, in vivo predictive dissolution, flow-through cell, virtual bioequivalence

## Abstract

**Background**: Candesartan cilexetil, a Biopharmaceutics Classification System (BCS) II prodrug, demonstrates compromised bioavailability attributable to its limited aqueous solubility coupled with P-glycoprotein (P-gp)-mediated efflux and hepatic first-pass metabolism, thereby introducing complexities in generic drug bioequivalence assessments. With the rapid advancement of computational technologies, the integration of biorelevant dissolution methodologies with physiologically based pharmacokinetic (PBPK) modeling is emerging as a transformative paradigm in advancing bioequivalence evaluation strategies for generic drug products. This study presents a GastroPlus^®^-driven framework integrating in vivo predictive dissolution (IPD) and virtual bioequivalence (VBE) to evaluate the quality consistency of generic candesartan cilexetil tablets. **Methods**: By developing an oral PBPK model in GastroPlus^®^, we established an IPD method using a phosphate-buffer-based flow-through cell dissolution apparatus. In vitro dissolution profiles of generic tablets from four manufacturers were measured and incorporated into the model to perform VBE simulations. **Results**: The results demonstrated that only the product from Company A achieved virtual bioequivalence with the reference product, aligning with real-world quality consistency assessments. **Conclusions**: The proposed framework exhibited robust predictive capability, bridging in vitro dissolution data to in vivo bioequivalence outcomes, thereby offering a cost-effective and efficient strategy for formulation optimization and preclinical bioequivalence evaluation of generic drugs.

## 1. Introduction

Dissolution testing remains a cornerstone of oral solid dosage form development, enabling rational formulation screening, manufacturing process refinement, and quality assurance across production batches [1]. Its role has expanded under modern regulatory paradigms, where dissolution profiles increasingly serve as surrogates for therapeutic equivalence assessments—particularly when in vitro–in vivo correlations (IVIVC) exist [2]. Nevertheless, conventional bioequivalence (BE) studies continue to depend on costly and ethically challenging clinical trials [3]. This limitation proves especially acute for Biopharmaceutics Classification System (BCS) II drugs (low solubility/high permeability), where dissolution-driven absorption variability complicates equivalence predictions [4].

In recent years, the rapid advancement of computer technology has provided many new ideas for drug research and development [5]. One example is pharmacokinetic studies integrating in vivo predictive dissolution (IPD) with physiologically based pharmacokinetic (PBPK) models. These approaches integrate in vitro dissolution data with in vivo absorption kinetics to simulate drug absorption and bioequivalence profiles, effectively circumventing ethical dilemmas while enhancing the scientific rigor of therapeutic equivalence assessments [6,7,8,9,10]. Notably, the flow-through cell system coupled with dynamic pH modulation has demonstrated exceptional accuracy in predicting dissolution for BCS II drugs [11,12,13,14]. The advantages of the flow-through cell lie in its capability to dynamically adjust the dissolution medium composition and flow rate during experiments. For dosage forms prone to floating, this method secures the sample at the center of the flow cell to ensure full contact with the dissolution medium. In the case of poorly soluble drugs, the open-loop configuration of the flow-through cell theoretically allows unlimited volumes of dissolution medium, making it superior to basket or paddle methods in maintaining sink conditions. Furthermore, by integrating fluid dynamics with biorelevant media (e.g., simulating gastrointestinal pH gradients), the flow-through cell effectively bridges IVIVC for challenging compounds [11]. When conducting dissolution testing, the selection of appropriate dissolution media is critical. According to the *Chinese Pharmacopoeia* (ChP) [15], the media should be specified in the individual monograph for each drug product, typically including: water (deaerated), 0.01~0.1 mol·L^−1^ hydrochloric acid (HCl) solution, and pH-adjusted buffer solutions (e.g., phosphate or acetate buffers). In contrast, the United States Pharmacopeia (USP) [16] mandates the use of standardized media such as pH 1.2 HCl solution, pH 4.5 acetate buffer, and pH 6.8 phosphate buffer. Additionally, surface-active agents (e.g., sodium dodecyl sulfate, SDS) may be incorporated into the media to enhance solubility for poorly water-soluble drugs if necessary.

Commercial software platforms, including GastroPlus^®^, PK-Sim^®^, Stella^®^, and GI-Sim^®^, now enable comprehensive simulations of drug absorption, distribution, metabolism, and excretion (ADME). Among these, GastroPlus^®^ integrates computational models of pharmacokinetics and pharmacodynamics while enabling the construction of ADME models to simulate drug behavior under diverse administration routes [17,18,19]. Through PBPK modeling, GastroPlus^®^ facilitates personalized simulations by accounting for interindividual variability [20,21,22], thereby supporting clinical dose optimization, drug safety evaluation, and regulatory compliance [23,24,25,26,27,28]. The multifaceted strengths of GastroPlus^®^ underpin its broad applicability. Primarily, it incorporates a comprehensive database of drug physicochemical and biopharmaceutical parameters, ensuring robust model foundation. Secondly, its computational framework spans the entire drug disposition cascade, enabling holistic simulation of in vivo drug behavior. Moreover, the software’s flexibility allows customized parameter adjustments to meet specific research objectives. For example, when investigating pharmacokinetics in special populations (e.g., pediatric, geriatric, or hepatorenal impairment cohorts), users can adjust physiological and disposition parameters to predict drug responses, thereby informing precision medicine strategies. This personalized modeling capability enables GastroPlus^®^ to play a pivotal role throughout drug development phases, ranging from early-stage drug design to late-phase clinical trial planning and regulatory submissions, thereby substantially enhancing both the efficiency and success rate of pharmaceutical research and development.

Candesartan, a selective angiotensin II receptor blocker predominantly administered as its prodrug candesartan cilexetil, is a first-line antihypertensive agent also indicated for cardiovascular conditions such as congestive heart failure [29,30]. However, its therapeutic potential is hindered by poor aqueous solubility (pKa 6.0), pronounced P-glycoprotein (P-gp)-mediated efflux, and extensive hepatic first-pass metabolism, collectively limiting oral bioavailability to 14% [31,32]. Marketed as Blopress^®^ by Takeda Pharmaceutical Co. (Tokyo, Japan), candesartan cilexetil tablets—a BCS II drug characterized by low solubility and high permeability—exhibit dissolution-limited absorption, making them an ideal candidate for establishing IVIVC [33,34].

**Figure 1:**
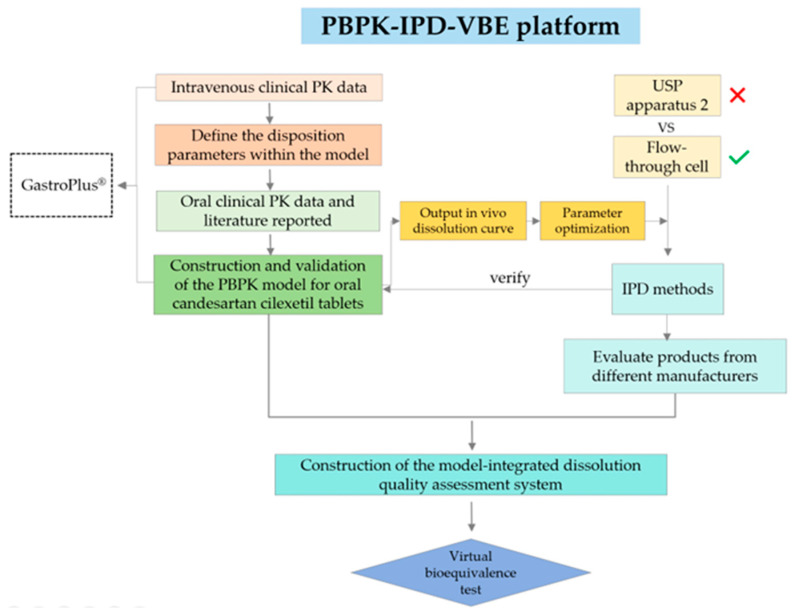
Framework of this research.

In this study, candesartan cilexetil tablets were selected as a model drug to develop a biorelevant flow-through cell dissolution method, construct a PBPK oral absorption model via GastroPlus^®^ for translating in vitro dissolution data into in vivo release kinetics, and perform VBE analysis. This integrated framework establishes a cost-efficient strategy to advance formulation development and therapeutic equivalence evaluations for BCS II drugs, addressing critical challenges in current regulatory practices (Figure 1).

## 2. Results and Discussion

### 2.1. Methodological Experiments

For the in vitro dissolution testing requiring analytical quantitation, method validation studies were conducted on the established High-Performance Liquid Chromatography (HPLC) analytical procedure in accordance with pharmacopeial requirements. The calibration curve demonstrating excellent linear correlation (y = 0.5483x − 0.0009, R^2^ = 1.0000) was established for candesartan cilexetil over the concentration range of 0.010 to 12.077 μg·mL^−^^1^, with the peak area showing proportional response to analyte concentration. The concentration of the limit of quantification (LOQ) was 0.010 μg·mL^−^^1^, and the blank excipient showed no significant interference. The recovery rates in different dissolution media are all between 98.1% and 99.2%, and the precision RSD (*n* = 6) is between 0.11% and 0.26%, which meets the requirements [15,35] for accuracy and precision. These results confirm the analytical procedure’s suitability for dissolution testing of candesartan cilexetil tablets.

### 2.2. USP Apparatus 2 Dissolution Test

**Figure 2:**
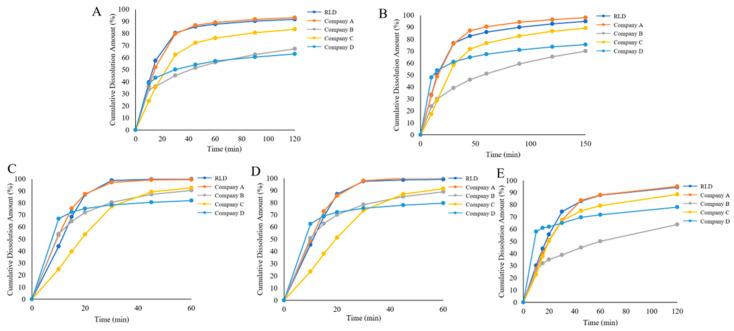
Dissolution profile comparison between the RLD and generic drug product of candesartan cilexetil tablets in different dissolution media. (**A**) pH 1.0 hydrochloric acid solution (1.0% Tween 20), (**B**) pH 4.5 acetate buffer (1.0% Tween 20), (**C**) pH 6.5 phosphate buffer (0.25% Tween 20), (**D**) pH 6.5 phosphate buffer (0.35% Tween 20), and (**E**) water (1.0% Tween 20).

In the USP apparatus 2 dissolution testing of candesartan cilexetil tablets, we conducted comparative analysis between the reference listed drug (RLD) and generic products (Figure 2). Dissolution profiles were statistically evaluated using the f2 similarity factor method (Table 1). The analysis revealed marked disparities between generic formulations from a certain company (notably Company D and B) and the RLD. Products of Company B, D, and C demonstrated lower cumulative dissolution. Company D’s product exhibited an initial rapid dissolution followed by a slowdown, while Company C’s product consistently showed slower dissolution rates compared to other formulations. Although Company B’s product displayed dissolution kinetics similar to the reference product within the first 10 min, a subsequent reduction in dissolution rate was observed. The f2 similarity factor analysis indicated that only Company A’s product exhibited dissolution behavior comparable to the reference product across all dissolution media.

### 2.3. Construction of GastroPlus^®^ Model

#### 2.3.1. Intravenous Prediction Model Results

**Figure 3:**
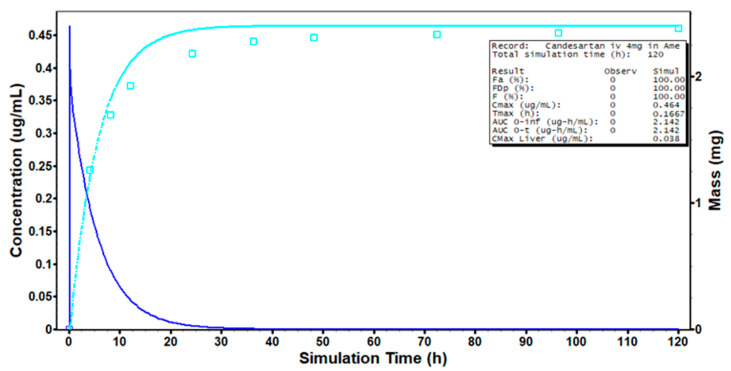
Prediction results of candesartan American intravenous PBPK model.

We incorporated literature-reported intravenous PK data of candesartan into the GastroPlus^®^ disposition model by setting corresponding clinical parameters for PK profile simulation. Observed data points represent urinary excretion amounts of candesartan, while simulated curves depict plasma concentrations (dark blue) and urinary excretion levels (light blue) (Figure 3). As candesartan is a P-glycoprotein (P-gp) substrate, it undergoes minimal metabolic clearance, with the majority of elimination occurring via biliary excretion. So, in the intravenous PBPK model, hepatic clearance was set to 0.748 L·h^−1^ (40% of total clearance) and renal clearance to 1.12 L·h^−1^ (60% of total clearance), resulting in a total systemic clearance of 1.868 L·h^−1^ (about 0.37 mL·min^−1^·kg^−1^), consistent with literature values. The calculated volume of distribution (10.479 L) aligned with literature-reported data (0.13 L·kg^−1^ × 84.25 kg). The model accurately predicted urinary drug excretion patterns, with renal elimination proportion reflecting candesartan’s in vivo clearance characteristics. Note that plasma PK curve comparison was omitted due to unavailable concentration–time data in the literature, though clearance and volume parameters remained literature-consistent. These results validate the accuracy of the Lukacova (Rodgers–Singh) method for predicting candesartan’s distribution. Consequently, the oral PBPK model of candesartan cilexetil adopted identical methodology.

#### 2.3.2. Oral Prediction Model Results

**Figure 4:**
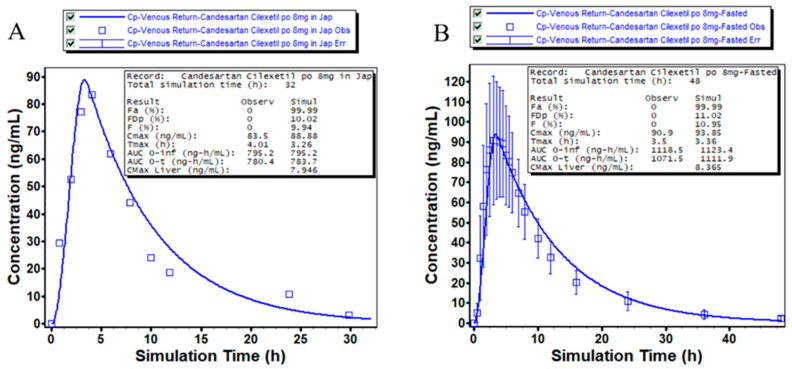
PBPK model predictions of 8 mg candesartan cilexetil tablets administered via preprandial oral route across ethnic populations. (**A**) Japanese and (**B**) Chinese.

Based on PK data from the regulatory submission documents of candesartan cilexetil tablets from Takeda and actual measurement data, we developed and validated PBPK models for 8 mg fasted oral administration in both Japanese and Chinese populations. Both models utilized the Opt logD Model SA/V 6.1 ASF model, with comparable intestinal first-pass effects but distinct total clearance values, while maintaining consistent hepatic (40%) and renal (60%) contributions to total clearance. Predictive accuracy (PE%) was calculated by comparing simulated and observed PK profiles to comprehensively evaluate the model’s ability to characterize absorption, distribution, and metabolism processes (Figure 4).

For all PK parameters in the 8 mg oral dose models, PE% values remained below 20% (Table 2), confirming the reliability of the physicochemical parameters, absorption model, disposition parameters, and computational algorithms in reflecting the in vivo behavior of candesartan cilexetil across ethnic populations.

### 2.4. In Vivo Dissolution and Absorption Evaluation Based on GastroPlus^®^

**Figure 5:**
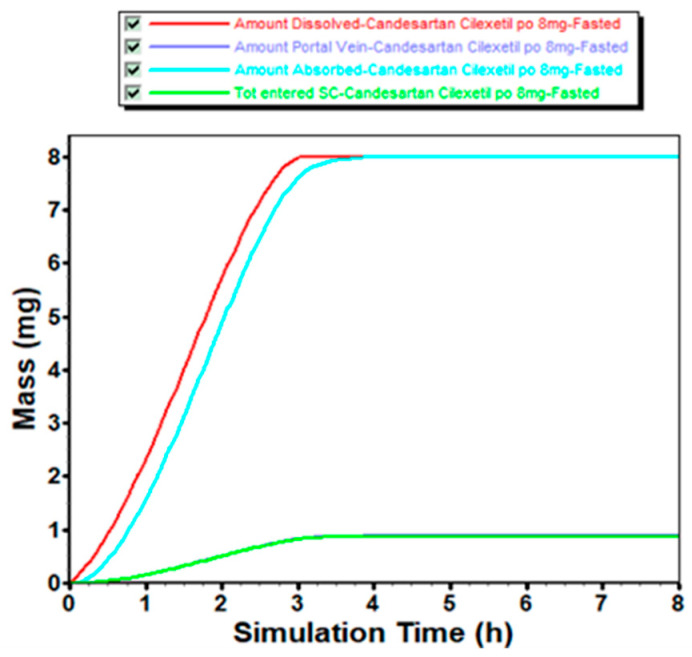
PBPK-model-predicted pharmacokinetic profiles of 8 mg candesartan cilexetil tablets following fasted oral administration in Chinese.

Using the established PBPK model for fasted oral administration of 8 mg candesartan cilexetil in Chinese subjects, we predicted the corresponding in vivo dissolution and absorption profiles (Figure 5). The results showed gradual drug dissolution in vivo, achieving complete dissolution within approximately 3 h after dosing. The dissolved drug exhibited rapid and complete transcellular absorption into enterocytes, confirming candesartan’s high permeability and indicating that permeability does not constitute the rate-limiting step in systemic absorption. This mechanistic understanding supports the feasibility of establishing IVIVC for dissolution behavior. By aligning in vitro dissolution methods with the predicted in vivo profiles, we further validated the utility of such correlations for predicting pharmacokinetics and conducting VBE assessments. These findings underscore the potential of leveraging IPD to streamline formulation development and bioequivalence evaluation.

**Figure 6:**
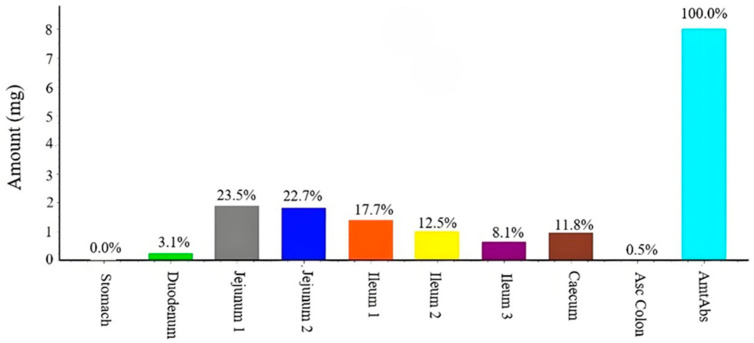
The absorption percentages of 8 mg candesartan cilexetil tablets in different intestinal segments after oral administration before meals in Chinese.

Furthermore, our investigation of in vivo absorption characteristics quantified the regional absorption percentages of candesartan cilexetil tablets across intestinal segments (Figure 6). During absorption, candesartan cilexetil undergoes conversion to candesartan, a known P-gp substrate. While the current model does not explicitly simulate P-gp-mediated efflux effects on candesartan, this transport mechanism was indirectly accounted for through intestinal first-pass effect parameterization. Simulation results under fasted conditions indicate complete absorption of candesartan cilexetil, with primary absorption sites localized to the jejunum, ileum, and cecum. However, approximately 90% of absorbed candesartan undergoes P-gp-dependent efflux back into the intestinal lumen, ultimately being excreted in the feces. This extensive enteric recycling results in an absolute bioavailability of approximately 11%.

### 2.5. Establishment of In Vivo Predictive Dissolution Method

**Figure 7:**
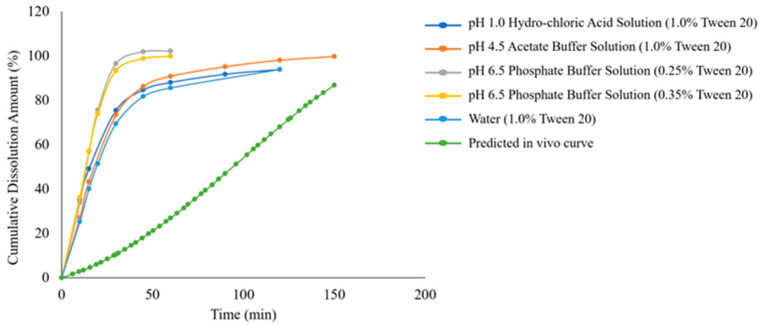
Comparative analysis between the in vivo release profile of the RLD and in vitro dissolution profiles obtained via USP apparatus 2 under varied biorelevant media conditions.

**Figure 8:**
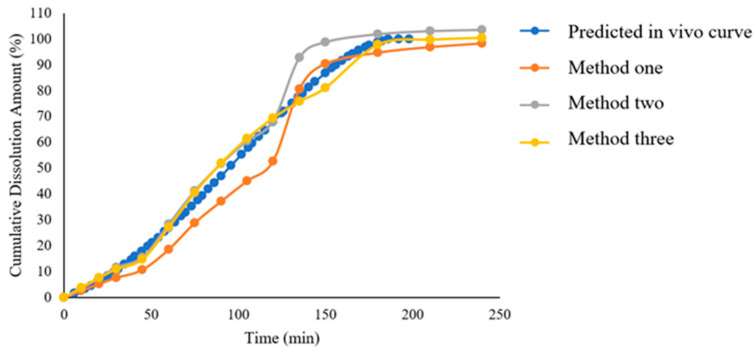
Comparative analysis of dissolution profiles under varied flow-through cell conditions.

**Figure 9:**
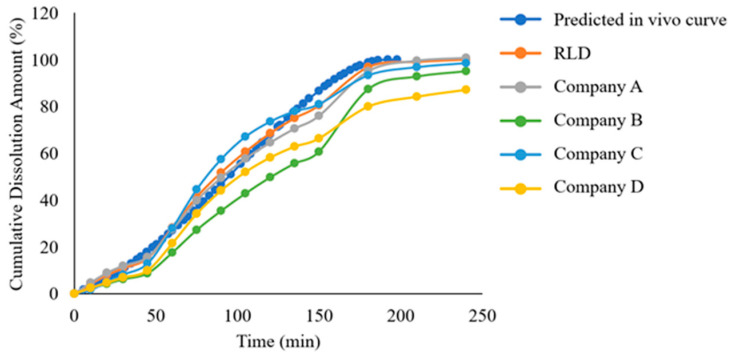
Dissolution profile characterization of candesartan cilexetil tablets (RLD vs. generic drugs) using the flow-through cell.

We compared the in vivo dissolution profile of the reference product predicted by GastroPlus^®^ software (version 9.5) simulation with the in vitro dissolution curve obtained using the USP apparatus 2 (Figure 7). The results demonstrated that candesartan cilexetil tablets exhibited a slow release process in vivo, with approximately 87% released at 150 min, and the dissolution rate was significantly lower than that observed in the in vitro dissolution curve. This indicates that, although the paddle method can differentiate product quality under different dissolution media to some extent, its insufficient biorelevance prevents it from reflecting the actual in vivo dissolution behavior of candesartan cilexetil tablets. Therefore, based on the in vivo release profile predicted by the GastroPlus^®^ model, we conducted dissolution testing using the more biorelevant flow-through cell method. By adjusting factors such as flow rate and pH gradient variations to optimize the alignment between the RLD’s in vitro dissolution profile and the predicted in vivo release profile, the methodological exploration is detailed in Table 3, with the resulting curves shown in Figure 8. The results demonstrated that under method-three conditions, the RLD exhibited the closest correlation between in vitro dissolution profiles and in vivo release curves, thus method three was selected as the discriminatory dissolution method for subsequent comparative dissolution testing of generic drug products from four companies (Figure 9).

### 2.6. Virtual Bioequivalence Simulation

**Figure 10:**
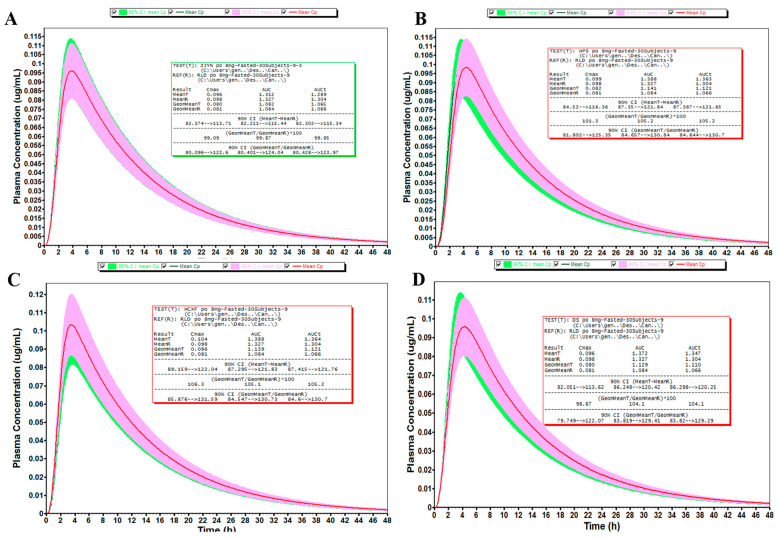
Predicted VBE results of generic drug products. (**A**) Company A, (**B**) Company B, (**C**) Company C, (**D**) Company D.

The in vitro dissolution profiles of the RLD and each generic formulation obtained from the experiments were incorporated into PBPK models to conduct virtual bioequivalence simulations for candesartan cilexetil tablets (Figure 10). The population simulation involved 30 virtual subjects, with bioequivalence determined by whether the 90% confidence intervals (CI) of the geometric mean ratios for Cmax, AUC, and AUC_t_ fell within 80–125% of the RLD. The virtual bioequivalence simulation results revealed that only the product from Company A demonstrated virtual bioequivalence to the RLD. Furthermore, market investigation confirmed that Company A was the sole company whose product had passed the national quality consistency evaluation, aligning with the simulation outcomes. Detailed results are summarized in Table 4. Previous studies [32] have demonstrated the utility of flow-through cell apparatus in developing predictive dissolution methods for candesartan cilexetil, where deconvolution-derived absorption profiles were employed to establish IVIVC models. In contrast, our approach leverages GastroPlus^®^-based simulations to optimize dissolution conditions by aligning in vitro release profiles with physiologically informed in vivo prediction curves. Following parameter refinement, the validated dissolution data were integrated into GastroPlus^®^ to perform VBE simulations. While conventional IVIVC methodologies rely on statistical correlations between dissolution and absorption, they often overlook physiological variabilities such as gastrointestinal motility, pH gradients, and transporter-mediated processes. The PBPK-IPD-VBE platform introduced in this study addresses these limitations by mechanistically incorporating formulation properties and patient-specific physiological factors, thereby enabling robust predictions under diverse clinical scenarios. This advancement holds particular promise for complex generics where traditional IVIVC frameworks face challenges. Furthermore, as PBPK databases and validation frameworks continue to expand—particularly for low-solubility, variable-permeability (BCS II/IV) compounds like candesartan cilexetil—the integration of in silico tools into bioequivalence assessments is anticipated to become a cornerstone of modern generic drug development.

## 3. Materials and Methods

### 3.1. Materials

Acetonitrile (HPLC grade) was purchased from Meck (Darmstadt, Germany). Sodium chloride, sodium acetate anhydrous, sodium hydroxide, potassium dihydrogen phosphate, and phosphoric acid (analytical grade) were purchased from Sinopharm Chemical Reagent Co., Ltd. (Shanghai, China). Acetic acid (analytical grade) was purchased from Lingfeng Chemical Reagent Co., Ltd. (Shanghai, China). Hydrochloric acid (analytical grade) was purchased from Hannuo Chemical Technology Co., Ltd. (Zhejiang, China). Tween 20 (analytical grade) was purchased from Wako Pure Chemical Industries, Ltd. (Osaka, Japan).

The reference standard of candesartan cilexetil (batch number: 100685-201903, 99.8% purity) was obtained from the National Institutes for Food and Drug Control (Beijing, China).

The reference products of candesartan cilexetil tablets were obtained from Tianjin Takeda Pharmaceuticals Co., Ltd. (Tianjin, China). The generic drugs were obtained from Company A (batch number: 1473A), Companies B (batch number: 2005126), Company C (batch number: 200503), and Company D (batch number: 200401).

### 3.2. HPLC Conditions

The analysis was performed on the LC-20ADXR liquid chromatography system from Shimadzu (Kyoto, Japan).

The quantification of candesartan was analyzed by HPLC-UV using a Kromasil 100-5 C18 column (5 μm, 4.6 × 50 mm) set at the controlled temperature of 35 °C. The mobile phase consisted of acetonitrile–glacial acetic acid–water (77:1:23, *v*/*v*/*v*). The flow rate was 1.0 mL/min, with an injection volume of 20 μL, and the detection wavelength was set at 254 nm.

### 3.3. Method Validation

Approximately 50 mg of candesartan cilexetil reference standard was accurately weighed and transferred to a 250 mL volumetric flask. The standard was dissolved in ethanol (HPLC grade) via ultrasonication, diluted to volume with ethanol, and homogenized to yield a stock solution (200 μg·mL^−1^). A working solution (8 μg·mL^−1^) was prepared by transferring 2.0 mL of the stock solution to a 50 mL volumetric flask and diluting with dissolution medium.

Calibration standards were prepared at different concentrations ranging from 0.010 μg·mL^−1^ to 12.077 μg·mL^−1^ through serial dilution. A calibration curve was constructed by plotting the analyte peak area against nominal concentrations, with linear regression analysis performed using the least squares method. The LOQ was defined as the lowest validated concentration meeting acceptable accuracy and precision criteria.

According to the analytical method validation guidelines of the ChP [15], samples at the same concentration (equivalent to 100% concentration level) were used to evaluate the accuracy and precision (*n* = 6). The recovery (%) and RSD (%) for the known spiked amount was calculated.

The calculation formula for recovery rate is as follows:

(1) $$Recovery=\frac{Measured\;concentration\;in\;spiked\;sample}{Nominal\;spike\;concentration}\times 100\%$$

Using a blank excipient solution prepared from Company A’s blank excipients, the interference of the excipients on the test results was examined.

### 3.4. In Vitro Dissolution Tests

A comparative evaluation was conducted to assess the predictive capacity of the flow-through cell versus the USP apparatus 2 in characterizing the in vivo dissolution behavior of candesartan cilexetil tablets. The in vitro dissolution data derived from the flow-through cell were subsequently integrated into a PBPK model for mechanistic analysis of drug absorption dynamics.

#### 3.4.1. USP Apparatus 2

The dissolution test of USP apparatus 2 was performed on the 709-DS automatic dissolution system from Agilent (Santa Clara, CA, USA).

Dissolution profiles were evaluated using USP Apparatus 2 under the following conditions: 900 mL of dissolution media (compositions detailed in Table 5), temperature maintained at 37.0 ± 0.5 °C, and agitation speed of 50 rpm. Twelve replicates per batch were analyzed and samples were continuously filtered through a 0.45 μm membrane filter and quantified via HPLC analysis (*n* = 12 for each batch).

#### 3.4.2. Flow-Through Cell

The dissolution test of the flow-through cell was performed on the SOTAX CE 7smart system from sotax (Basel, Switzerland).

The parameters were configured as detailed in Table 6. The experimental setup consisted of a ruby bead fixed at the bottom of a 12 mm cell, followed by standardized scoop-loading of 1 mm glass beads. Tablets were secured in sample holders, with the cell assembly capped by a GF/D (2.7 μm) and GF/F (0.7 μm) glass microfiber filter system containing defatted cotton purchased from Glasai Life Sciences (Shanghai, China) Co., Ltd. (Shanghai, China). Six parallel experiments (*n* = 6) were simultaneously performed under controlled temperature conditions (37 ± 0.5 °C). Samples were continuously filtered through a 0.45 μm membrane filter and analyzed by HPLC using a validated method consistent with USP apparatus 2 specifications.

### 3.5. Construction of PBPK Model

The PBPK model of candesartan cilexetil was developed using commercially available PBPK modeling software GastroPlus^®^ (version 9.5, Simulations Plus, Inc., Lancaster, CA, USA) and Digit literature data extraction software (version 1.0.4, Simulation Plus, Inc., Lancaster, CA, USA).

Physicochemical and biopharmaceutical parameters for model construction were integrated from multiple sources, including literature-reported data, experimentally measured values, software-default parameters, and quantitative structure-based predictions derived from candesartan cilexetil and its active metabolite candesartan (Table 7).

The in vivo dissolution process of candesartan cilexetil tablets was examined using the Johnson dissolution model, with the formula as follows:

(2) $$\begin{array}{c} \frac{dM_{d}}{dt}=\frac{D}{\rho h\gamma_{t}}\times \frac{\left(1+2s\right)}{S}\times \left(C_{s}-C_{l}\right)\times M_{u,t} \end{array}$$

where *D* is the diffusion coefficient, *ρ* is the particle density, *r* is the particle radius, *h* is the diffusion layer thickness of the dissolution process, *s* is the shape factor (the ratio of the particle length to diameter), *C* is the drug solubility, and *M_u,t_* is the amount of undissolved drug.

The in vivo absorption of candesartan cilexetil tablets was simulated by integrating GastroPlus^®^’s mechanistic absorption models with physiological gastrointestinal parameters. The governing equations are expressed as:

(3) $$\frac{dM_{abs,i}}{dt}={ASF}_{trans,i}\times P_{trans,i}\times V_{lum,i}\times \left({c\left(t\right)}_{lum,i}-{c\left(t\right)}_{entU,i}\right)\;\;\;\;\;\;\;\;\;+{ASF}_{para,i}\times P_{para,i}\times V_{lum,i}\times \left({c\left(t\right)}_{lum,i}-{c\left(t\right)}_{pvU}\right)$$

where *ASF_trans,i_* and *ASF_para,i_* are absorption-scale factors for transcellular and paracellular diffusion, theoretical ASF is calculated as the surface area-to-volume ratio (equals 2 divided by R_i_, where R_i_ represents the radius of intestinal segment i), *P_trans,i_* and *P_Para,i_* are effective permeability coefficients for transcellular and paracellular pathways, *V_lum,i_* is luminal volume in gastrointestinal compartment, *C(t)_lum,i_* is time-dependent drug concentration in intestinal lumen of compartment, *C(t)_entU,i_* is unbound drug concentration in enterocytes of compartment, and *C(t)_pvU_* is unbound drug concentration in hepatic portal vein.

The gastrointestinal physiological model was implemented using the Advanced Compartmental Absorption and Transit (ACAT) framework. The Absorption Scale Factor (ASF) model employed the Opt logD Model SA/V 6.1 (default configuration), with key physiological parameters as detailed in Supplementary Materials.

Candesartan cilexetil undergoes rapid and complete hydrolysis to candesartan during gastrointestinal absorption, with only the active metabolite entering systemic circulation. Consequently, the physicochemical parameters of candesartan were utilized in conjunction with tissue-specific physiological parameters to simulate its distribution kinetics. The steady-state volume of distribution was estimated using the Poulin method, incorporating tissue volumes and tissue-to-plasma partition coefficients:

(4) $$\begin{array}{c} V_{ss}=V_{p}+V_{e}\times E:P+\sum V_{t}\times {Kp}_{t}\times \left(1-{ER}_{t}\right) \end{array}$$

where *V_p_* is plasma volume, *V_e_* is erythrocyte volume, *E:P* is erythrocyte-to-plasma concentration ratio (calculated from blood-to-plasma ratio B/P and hematocrit), *V_t_* is tissue volume, *Kp_t_* is tissue-to-plasma partition coefficient (predicted via the Lukacova method, also known as Rodgers–Singh methodology), and *ER_t_* is tissue-specific uptake rate.

The candesartan cilexetil intravenous and oral model was developed by replicating literature-reported [41,42] administration protocols (e.g., identical route, dosage, and subject demographics) as detailed in Table 8. Model validation involved comparative analysis between simulated PK profiles and observed clinical data, with particular emphasis on optimizing clearance (CL) and volume of distribution (Vd) parameters. Subsequently, human oral PK profiles from published studies were incorporated into the GastroPlus^®^ disposition module through clinical parameter alignment. This enabled simulation-based evaluation of model predictive accuracy for in vivo dissolution-absorption processes, culminating in definitive characterization of ADME parameters and associated computational algorithms.

Leveraging pharmacokinetic data from the reference formulation, the Johnson dissolution model was implemented to simulate IPD profiles in Chinese populations. The derived in vivo dissolution–absorption correlation matrix informed development of clinically relevant dissolution testing methodologies.

### 3.6. Virtual Bioequivalence Evaluation

Virtual bioequivalence assessment serves as a critical tool for demonstrating pharmaceutical quality and therapeutic performance throughout the product lifecycle, while supporting formulation optimization through mechanistically grounded predictions.

Virtual bioequivalence trials provide scientific evidence for the safety and efficacy of pharmaceutical products, ensuring quality and performance throughout the entire product lifecycle and offering support for the development of drug formulations. By integrating in vitro dissolution profiles into the established PBPK models, we simulated in vivo PK curves using the Weibull equation and compared them with experimentally measured PK profiles. The simulation involved a virtual population of 30 subjects over a 48 h period.

The Weibull equation is as follows:

(5) $$\begin{array}{ll} \%DoseRelease & =Max\times (1-f_{1}\exp\left[\frac{{-\left(t-T\right)}^{b1}}{A_{1}}\right]-f_{2}\exp\left[\frac{{-\left(t-T\right)}^{b2}}{A_{2}}\right]\\ & -f_{3}\exp\left[\frac{{-\left(t-T\right)}^{b3}}{A_{3}}\right] \end{array}$$

where *Max* is the total dissolution percentage, *T* is the lag time (h), *f*1, *f*2, and *f*3 are the dissolution fractions of the 1st, 2nd, and 3rd phases (*f*_1_ + *f*_2_ + *f*_3_ = 1), *b*1, *b*2, and *b*3 are the shape factors for the 1st, 2nd, and 3rd phases, and *A*_1_, *A*_2_, and *A*_3_ are the corresponding phase time scales.

## 4. Conclusions

The above experiments demonstrated that the flow-through cell method could better simulate in vivo conditions. Based on this, we developed an IPD method for generic drug consistency evaluation. By integrating this method with a PBPK model established using GastroPlus^®^, we successfully simulated the absorption, distribution, and metabolism of candesartan cilexetil in different populations. Furthermore, virtual bioequivalence results indicated that only products of Company A exhibited bioequivalence with the reference product. In conclusion, the integrated PBPK-IPD-VBE platform established in this study provides a robust framework for simulating dissolution, absorption, distribution, and metabolism of candesartan cilexetil tablets in Chinese populations. This approach enhances quality consistency evaluation, formulation development, and preclinical bioequivalence prediction, ultimately accelerating generic drug development cycles and reducing research and development costs. While European and American PK data were not available in the current study, future work will focus on model refinement and validation for these populations through prospective clinical data collection.

## Figures and Tables

**Table 1:** Summary of f2 factor results.

Manufacturer	pH 1.0 Hydrochloric Acid Solution (1.0% Tween 20)	pH 4.5 Acetate Buffer Solution (1.0% Tween 20)	pH 6.5 Phosphate Buffer Solution (0.25% Tween 20)	pH 6.5 Phosphate Buffer Solution (0.35% Tween 20)	Water (1.0% Tween 20)
A	73	80	74	58	69
B	29	28	48	49	28
C	38	40	26	28	39
D	32	41	44	41	39

**Table 2:** Summary data of PK parameters for predicted results versus measured results, and assessment of prediction accuracy.

	Category	C_max_ (ng·mL^−1^)	T_max_ (h)	AUC_0-inf_ (ng·h·mL^−1^)	AUC_0-t_ (ng·h·mL^−1^)
Candesartan Cilexetil po 8 mg in Japanese	Literature Value	83.5	4.01	795.2	780.4
	Predicted Value	88.88	3.26	795.2	783.7
	PE% ^1^	6.44	18.70	0.00	0.42
Candesartan Cilexetil po 8 mg in Chinese	Measured Value	90.9	3.5	1118.5	1071.5
	Predicted Value	93.85	3.36	1123.4	1111.9
	PE% ^1^	3.25	−4.00	0.44	3.77

^1^ PE% = |Predicted-Measured|/Measured × 100.

**Table 3:** Flow-through cell method exploration parameters.

Selected Method	Flow Rate (mL·min^−1^)	Medium	Sampling Time (min)
Method One (Open Loop)	4	pH 1.2 Hydrochloric Acid Solution (0.2% Tween 20)	10, 20
		pH 4.5 Acetate Buffer Solution (0.2% Tween 20)	30, 45
		pH 5.7 Phosphate Buffer Solution (0.2% Tween 20)	60, 75, 90, 105, 120
		pH 6.8 Phosphate Buffer Solution (0.3% Tween 20)	135, 150, 180, 210, 240
Method Two (Open Loop)	6	pH 1.2 Hydrochloric Acid Solution (0.2% Tween 20)	10, 20
		pH 4.5 Acetate Buffer Solution (0.2% Tween 20)	30, 45
		pH 5.7 Phosphate Buffer Solution (0.2% Tween 20)	60, 75, 90, 105, 120
		pH 6.8 Phosphate Buffer Solution (0.3% Tween 20)	135, 150, 180, 210, 240
Method Three (Open Loop)	6	pH 1.2 Hydrochloric Acid Solution (0.2% Tween 20)	10, 20
		pH 4.5 Acetate Buffer Solution (0.2% Tween 20)	30, 45
		pH 5.7 Phosphate Buffer Solution (0.2% Tween 20)	60, 75, 90, 105, 120, 135, 150
		pH 6.8 Phosphate Buffer Solution (0.3% Tween 20)	180, 210, 240

**Table 4:** Virtual bioequivalence prediction results.

		Company A	Company B	Company C	Company D
Cmax	90% CI (MeanT-MeanR)	82.37~113.71	84.52~116.36	89.16~122.04	82.05~113.62
	(GeomMeanT/GeomMeanR) × 100	99.09	101.3	106.3	98.67
	90% CI (GeomMeanT/GeomMeanR)	80.10~122.60	81.80~125.35	85.88~131.59	79.75~122.07
AUC	90% CI (MeanT-MeanR)	82.21~115.44	87.35~121.84	87.30~121.83	86.25~120.42
	(GeomMeanT/GeomMeanR) × 100	99.87	105.2	105.1	104.1
	90% CI (GeomMeanT/GeomMeanR)	80.40~124.04	84.66~130.84	84.55~130.73	83.82~129.41
AUC_t_	90% CI (MeanT-MeanR)	82.30~115.34	87.39~121.65	87.42~121.76	86.30~120.25
	(GeomMeanT/GeomMeanR) × 100	99.85	105.2	105.2	104.1
	90% CI (GeomMeanT/GeomMeanR)	80.43~123.97	84.64~130.70	84.60~130.70	83.82~129.29

**Table 5:** Parameter settings of USP apparatus 2.

Medium	Flow Rate (r·min^−1^)	Sampling Time (min)
Water (1.0% Tween 20)	50	10, 15, 30, 45, 60, 90, 120
pH 1.0 Hydrochloric Acid Solution (1.0% Tween 20)	50	10, 15, 30, 45, 60, 90, 120
pH 4.5 Acetate Buffer Solution (1.0% Tween 20)	50	10, 15, 30, 45, 60, 90, 120, 150
pH 6.5 Phosphate Buffer Solution (0.25% Tween 20)	50	10, 15, 20, 30, 45, 60
pH 6.5 Phosphate Buffer Solution (0.35% Tween 20)	50	10, 15, 20, 30, 45, 60

**Table 6:** Parameter settings of flow-through cell.

Medium	Flow Rate (mL·min^−1^)	Sampling Time (min)
pH 1.2 Hydrochloric Acid Solution (0.2% Tween 20)	6	10, 20
pH 4.5 Acetate Buffer Solution (0.2% Tween 20)	6	30, 45
pH 5.7 Phosphate Buffer Solution (0.2% Tween 20)	6	60, 75, 90, 105, 120, 135, 150
pH 6.8 Phosphate Buffer Solution (0.3% Tween 20)	6	180, 210, 240

**Table 7:** Parameter settings of candesartan cilexetil PBPK model.

Parameter	Value	Source
Molecular Weight	440.46 g·mol^−1^ (candesartan) 610.67 g·mol^−1^ (candesartan cilexetil)	
logP	3.42 (candesartan)	[36]
	7.1 (candesartan cilexetil)	[37]
pKa	1.45, 4.23 (candesartan)	[36]
	Acid: 6.0 (candesartan cilexetil)	[38]
Solubility (pH-solubility)	pH 1.0: 0.23 μg·mL^−1^ pH 4.5: 0.51 μg·mL^−1^ pH 6.5: 0.8 μg·mL^−1^ Water (pH 6.8): 1.4 μg·mL^−1^	[36]
Precipitation Time	900 s	Default Value
Diffusion Coefficient	0.63 × 10^−5^ cm^2^·s^−1^	Calculated based on the formula built in GastroPlus^®^
Particle Density	1.2 g·mL^−1^	Default Value
Particle Size	4 μm	[39]
Human Permeability	Human jejunal permeability coefficient: 3.6 × 10^−4^ cm·s^−1^	[40]
Whole Blood/Plasma Drug Concentration Ratio	0.68	Predicted Value from ADMET Predictor 9.5
Plasma Unbound Drug Fraction	1%	Instruction Manual: Candesartan is more than 99% bound to plasma proteins

**Table 8:** Basic setup of intravenous and oral PK model for candesartan.

Model	Dose (mg)	Population	Clearance Rate	Source
Candesartan Cilexetil iv 4 mg in American	4	American male (25 years old, 84.25 Kg body weight) under fasted conditions	/	Literature report
Candesartan Cilexetil po 8 mg in Japanese	8	Japanese male (30 years old, 62.57 Kg body weight) under fasted conditions	Liver: 0.4 L·h^−1^ Kidney: 0.6 L·h^−1^	Candesartan cilexetil tablet registration documents from Takeda
Candesartan Cilexetil po 8 mg in Chinese	8	Chinese male (30 years old, 63 Kg body weight) under fasted conditions	Liver: 0.312 L·h^−1^ Kidney: 0.468 L·h^−1^	Measured data

## Data Availability

The data presented in this study are available in this article.

## Supplementary Materials

The following are available online at https://www.mdpi.com/article/pharmaceuticals-18-00562/s1, Table S1: Accuracy and precision assessment results obtained by HPLC-UV; Figure S1: Physiological parameter diagram of candesartan cilexetil PBPK model.
