# Supplementary material for: From In Vivo Predictive Dissolution to Virtual Bioequivalence: A GastroPlus®-Driven Framework for Generic Candesartan Cilexetil Tablets
Source: Pharmaceuticals (Basel). 2025 Apr 11;18(4):562. doi: 10.3390/ph18040562 (PMC12030460; doi:10.3390/ph18040562)
Supplement: Supplementary file 1 [file pharmaceuticals-18-00562-s001.zip › pharmaceuticals-3566972-supplementary.pdf]

Table S1. Accuracy and precision assessment results obtained by HPLC-UV.

	pH 1.0 Hydrochloric Acid Solution (1.0% Tween 20)	pH 4.5 Acetate Buffer Solution (1.0% Tween 20)	pH 6.5 Phosphate Buffer Solution (0.25% Tween 20)	pH 6.5 Phosphate Buffer Solution (0.35% Tween 20)	Water (1.0% Tween 20)
1	98.1	98.3	99.2	98.8	98.7
2	98.6	98.6	98.8	99.1	98.9
3	98.8	98.5	99.0	99.1	98.6
4	98.8	98.4	98.6	98.6	98.6
5	98.6	98.6	98.8	99.1	98.8
6	98.7	98.7	99.2	99.0	98.7
Average Recovery Rate (%)	98.6	98.5	98.9	99.0	98.7
RSD (%)	0.26	0.15	0.23	0.21	0.11

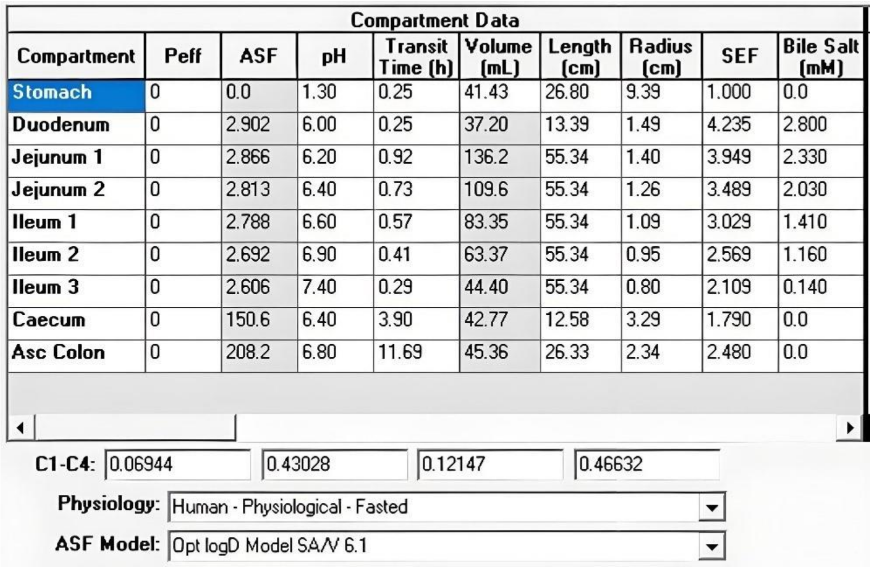

Compartment Data

Compartment	Peff	ASF	pH	Transit Time (h)	Volume (mL)	Length (cm)	Radius (cm)	SEF	Bile Salt (mM)
Stomach	0	0.0	1.30	0.25	41.43	26.80	9.39	1.000	0.0
Duodenum	0	2.902	6.00	0.25	37.20	13.39	1.49	4.235	2.800
Jejunum 1	0	2.866	6.20	0.92	136.2	55.34	1.40	3.949	2.330
Jejunum 2	0	2.813	6.40	0.73	109.6	55.34	1.26	3.489	2.030
Ileum 1	0	2.788	6.60	0.57	83.35	55.34	1.09	3.029	1.410
Ileum 2	0	2.692	6.90	0.41	63.37	55.34	0.95	2.569	1.160
Ileum 3	0	2.606	7.40	0.29	44.40	55.34	0.80	2.109	0.140
Caecum	0	150.6	6.40	3.90	42.77	12.58	3.29	1.790	0.0
Asc Colon	0	208.2	6.80	11.69	45.36	26.33	2.34	2.480	0.0

C1-C4: 0.06944 | 0.43028 | 0.12147 | 0.46632

Physiology: Human - Physiological - Fasted

ASF Model: Opt logD Model SA/V 6.1

Figure S1. Physiological parameter diagram of candesartan cilexetil PBPK model.
